# Principles of sample size calculation

**DOI:** 10.4103/0301-4738.71692

**Published:** 2010

**Authors:** Nithya J Gogtay

**Affiliations:** Department of Clinical Pharmacology, Seth GS Medical College and KEM Hospital, Parel, Mumbai, Maharashtra, India

**Keywords:** Alpha error, beta error, clinically meaningful difference, variability

## Abstract

In most areas in life, it is difficult to work with populations and hence researchers work with samples. The calculation of the sample size needed depends on the data type and distribution. Elements include consideration of the alpha error, beta error, clinically meaningful difference, and the variability or standard deviation. The final number arrived at should be increased to include a safety margin and the dropout rate. Over and above this, sample size calculations must take into account all available data, funding, support facilities, and ethics of subjecting patients to research.

In most areas in life, it is very difficult to work with populations. During elections for instance, news channels interview a few hundred people and predict results based on their choices. Similarly, in a factory manufacturing light bulbs, a few bulbs are chosen at random to assess their quality. Likewise, in research, while it is ideal to work with the entire population, it is almost impossible to do so. Hence researchers choose to work with samples. Sample size calculations enable researchers to draw strong robust conclusions from the limited amount of information and also permit generalization of results. It is however important to remember that since it is very difficult to predict the outcome of any clinical study or lab experiment, sample size calculations will always remain approximate.

The estimation of the minimum sample size required for any study is not a single unique method, but the concepts underlying most methods are similar. The determination of the sample size is critical in planning clinical research because this is usually the most important factor determining the time and funding to perform the experiment. In most studies, there is a primary research question that the researcher wants to investigate. Sample size calculations are based on this question. Sample size calculations must take into account all available data, funding, support facilities, and ethics of subjecting patients to research. The present paper outlines the principles of sample size calculation for randomized controlled trials (RCTs) with a few solved examples.

## Elements in Sample Size Calculation[1]

Sample size calculations begin with an understanding of the type of data and distribution we are dealing with. Very broadly, data are divided into quantitative (numerical) and categorical (qualitative) data. For the former, information on the mean responses in the two groups’ *u*1 and *u*2 are required as also the common standard deviation for the two groups. For categorical data, *p*1 and *p*2 or information on proportions of successes in the two groups is needed. This information is usually obtained either from the published literature, a pilot study, or at times guesstimated. The other two key components are the alpha and beta error. Because the estimated sample size represents the minimum number of subjects required for the study, a “safety factor” should be added. The size of the safety factor is again an educated guess. Additions for drop-outs/attrition during the course of the study should also be made. Apart from this, an understanding of whether the data are normally distributed (follows the Gaussian or bell-shaped curve) or otherwise is also needed.[2]

## Understanding of Key Terms[3]

The calculation of sample size based on power considerations requires that an investigator specify the points given below. The first three items are under the control of the investigator:

*The size of the effect that is clinically worthwhile to detect (d)*. This for numerical data is the difference between u1 and u2 for quantitative data and p1 and p2 for categorical data. This is also called the clinical meaningful difference, which will make the physician change his or her practice.

*The probability of falsely rejecting a true null hypothesis (α-error)*. This is also called the false positive error and is the probability of finding a difference where none exists. This error is perceived to be the more dangerous of the two errors, since it can impact clinical practice. It is also called the regulator’s error. The alpha error is linked to the *P*-value or probability value and is conventionally set at 5%.

*The probability of failing to reject a false null hypothesis (β-error)*. This is also called the false negative error and is the probability of NOT finding a difference when one actually exists. This is conventionally set either at 10% or 20% and is also called the investigator’s error.

*The standard deviation of the population being studied (SD or σ)*. This is the variability or spread associated with quantitative data.

Standard values of the alpha and beta error are given in the solved examples and can be found in most statistics books. The examples below can be solved by hand using simple or scientific calculators. The website and the pdf file, http://www.idfbridges.org/files/BRIDGES-sample-size-calculation-and-example-of-budget.pdf, provide an easy tool of how to use online sample size calculators.

## Few Solved Examples

- (A) Sample size for one mean, normal distribution
  $$n\;=\;\frac{{\left(Z_{\alpha }\;+\;Z_{\beta }\right)}^{2}\times \;\sigma^{2}}{d^{2}}\;$$
  *Problem*: An emergency medicine physician wants to know if the mean heart rate after a particular type of trauma differs from the healthy population rate of 72 beats/min. He considers a mean difference of 6 beats/min to be clinically meaningful. He also chooses 9.1 beats/min as the variation based on a previously published study. How many patients will be needed to carry out the study at 5% significance and 80% power?
  In this example, the following data are given to us:
  - the size of the effect that is clinically worthwhile to detect (*d*) = 6 beats/min
  - the probability of falsely rejecting a true null hypothesis (α) = 0.05, Z_α_ = 1.96
  - the probability of failing to reject a false null hypothesis (β) = 0.80, Z_β_ = 0.84
  the standard deviation of the population being studied (SD or σ) = 9.1 beats/min.
  $$n\;=\;\frac{{\left(1.96\;+\;0.84\right)}^{2}\times {\left(9.1\right)}^{2}}{{\left(6\right)}^{2}}\;$$
  n = 18
  or
  18 patients with a particular type of trauma need to be studied by the physician.
- (B) Sample size for two means, quantitative data
  $$n\;=\;\frac{{\left(Z_{\alpha }\;+\;Z_{\beta }\right)}^{2}\times \;\sigma^{2}}{d^{2}}\;$$
  where *d* = *u*1 - *u*2/2.
  *Problem*: A new treatment for hypertension is being compared with placebo. How many patients will be required at 90% power and 5% significance to detect an average difference of 5 mmHg between the Rx group and placebo group assuming a standard deviation (a measure of interpatient variability) to be 10 mm?
  In this example, the following data are given to us:
  - the size of the effect that is clinically worthwhile to detect (*d*) = 5 mm
  - the probability of falsely rejecting a true null hypothesis (α) = 0.05, Z_α_ = 1.96
  - the probability of failing to reject a false null hypothesis (β) = 0.80, Z_β_ = 1.282
  - the standard deviation of the population being studied (SD or σ) = 10 mm.
  Solution:
  $$n\;=\;\frac{2{\left(1.96\;+\;1.282\right)}^{2}}{{\left(d\right)}^{2}}\;$$
  where *d* = 5/10
  thus, *n* = 84 patients per group.
- (C) Sample size for two proportions, categorical data
  *The BASIL study-bypass versus angioplasty*: The statistical calculations were based on the 3-year survival value of 50% in the angioplasty and 65% in the bypass group. At 5% significance and 90% power, how many patients would be needed to detect a difference between the two groups? (Lancet 2005;366:1925-34).
  In this example, the following data are given to us:
  - the size of the effect that is clinically worthwhile to detect (*d*) = 15% or 0.15
  - the probability of falsely rejecting a true null hypothesis (*α*) = 0.05, Z_α_ = 1.96
  - the probability of failing to reject a false null hypothesis (*β*) = 0.80, Z_β_ = 1.282.
  $$n\;=\;\frac{2{\left(Z_{\alpha }\;+\;Z_{\beta }\right)}^{2}}{\left(d^{2}\right)}\;$$
  where, *d* is
  *p*1 – *p*2
  √*p* (1-*p*).
  And *p* = *p*1 + *p*2/2
  Solution:
  $$n\;=\;\frac{2{\left(1.96\;+\;1.282\right)}^{2}}{\left(0.03\;\times \;0.03\right)}\;$$
  *p*1 = 0.65, *p*2=.50, *p* = 0.575,
  i.e., there will be 233 patients per group.
  The sample size calculation should be done with the help of a statistician. However, the present article provides the basic understanding of the principles behind the sample size calculation. This would help in providing the required inputs to the clinicians while interacting with the statistician.
